# The Role of the Respiratory Therapy Team in the Treatment of Patients With Acquired Immunodeficiency Syndrome Complicated With Pneumocystis Pneumonia Undergoing Mechanical Ventilation

**DOI:** 10.3389/fpubh.2022.799159

**Published:** 2022-03-25

**Authors:** Yanmei Gu, Shuangmei Xi, Boxun Jin, Huimin Guo, Jiankun Yang, Xin Liu, Xiulian Wu, Lili Zhang, Guangming Li

**Affiliations:** ^1^Department of Critical Care Medicine, Beijing Youan Hospital, Capital Medical University, Beijing, China; ^2^Department of Nursing, Beijing Youan Hospital, Capital Medical University, Beijing, China

**Keywords:** respiratory care team, aids, PCP, nursing, mechanically ventilated patients

## Abstract

**Objective:**

To explore the role of the respiratory therapy team in the treatment of patients with acquired immunodeficiency syndrome (AIDS) complicated with pneumocystis pneumonia (PCP) undergoing mechanical ventilation.

**Methods:**

A retrospective cross-sectional study was conducted, including 60 patients with AIDS complicated with PCP undergoing mechanical ventilation in our hospital from June 2019 to July 2020. In the process of patient respiratory monitoring, hospital transport, ventilator withdrawal, airway management, various aerosol treatments and controlled oxygen therapy, patients were divided into the control group and the case group according to whether the respiratory therapy team was involved or not (30 in the control group, 25 males and five females; 30 in the case group, 24 males and six females). The baseline data, mechanical ventilation time, hospitalization time and hospitalization expenses of the two groups were compared.

**Results:**

There was no statistically significant difference in baseline data between the case and control groups (*P* > 0.05). Compared with the control group, the case group had significantly shorter mechanical ventilation times and average hospitalization lengths and the average expenses decreased, and the difference was statistically significant (*P* < 0.05).

**Conclusion:**

The participation of the respiratory therapy team in the mechanical ventilation treatment of patients with AIDS and PCP helps to shorten the mechanical ventilation time and the average length of hospitalization and reduce the hospitalization expenses of patients. It is expected to increase the cure rate of such patients and improve their prognosis.

## Introduction

Acquired immune deficiency syndrome (AIDS) is a chronic fatal disease usually result from infections by human immunodeficiency virus (HIV) among the immunocompromised patients. HIV infection refers to the toxic state of HIV after entering the human body, that is, HIV-infected people. HIV-infected people with serious clinical symptoms are called AIDS patients. China entered a rapid growth period in 1995 (1). PCP is one of the most familiar opportunistic infections as we known and is also the main reason of increased mortality among patients with AIDS (2). Patients with pulmonary infection caused by PCP account for about 50–60% of the population with AIDS and 85% of patients with AIDS have at least one PCP infection during their illness, and 25% of them died of PCP. PCP has a hidden clinical onset and rapid and dangerous disease development. If it is not treated in time, it can disable the immune system gradually and even the acute respiratory distress syndrome (ARDS) in a very short time, increasing mortality (3).

Mechanical ventilation can alleviate hypoxia, relieve respiratory muscle fatigue, reduce oxygen consumption of the body, improve hypoxemia in patients with respiratory failure and buy sufficient time to treat the primary disease. Respiratory care is an emerging health treatment discipline focusing on cardiopulmonary function support and rehabilitation (4) and has become a routine treatment for ICU patients in Europe and America. The progress of medical education and clinical medicine in China is still not perfect. There are <1,000 practitioners related to respiratory therapy (5), and the limited amout of professional respiratory therapists is insuffcient to meet the clinical needs (6). Respiratory therapy is mainly undertaken by doctors and nurses working in the respiratory department and ICU. With the emergence of a large number of new diagnoses and treatment technologies and more types of respirators used in clinics, the medical staff cannot master them all, and professional personnel is needed to manage, monitor and maintain them (7). Choosing senior nursing staff to become professional respiratory therapists after corresponding respiratory therapy training has become the choice of most hospitals in China (8–10).

Our hospital established a respiratory therapy team on 1 January 2020. This study analyses the role of the respiratory therapy team in the treatment of patients with AIDS complicated with PCP undergoing mechanical ventilation, wishing to offer a theoretical basis and clinical experience for therapeutic methods.

## Materials and Methods

### Subjects

Sixty patients with AIDS complicated with PCP undergoing mechanical ventilation admitted to our hospital from 1 June 2019 to 31 July 2020 were analyzed retrospectively. Inclusion criteria: 1. mechanically ventilated patients who met the diagnostic criteria for AIDS complicated with PCP; 2. patients who gave informed consent to this study and signed an informed consent form. Exclusion criteria: 1. patients complicated with severe heart, liver and kidney diseases; 2. patients who withdrew/transferred/discharged during the course; 3. patients who refused to sign informed consent; 4. none of the above, but the patient was temporarily unable to sign the informed consent due to coma or other reasons, and there was no legal representative to sign instead.

**Figure 1:**
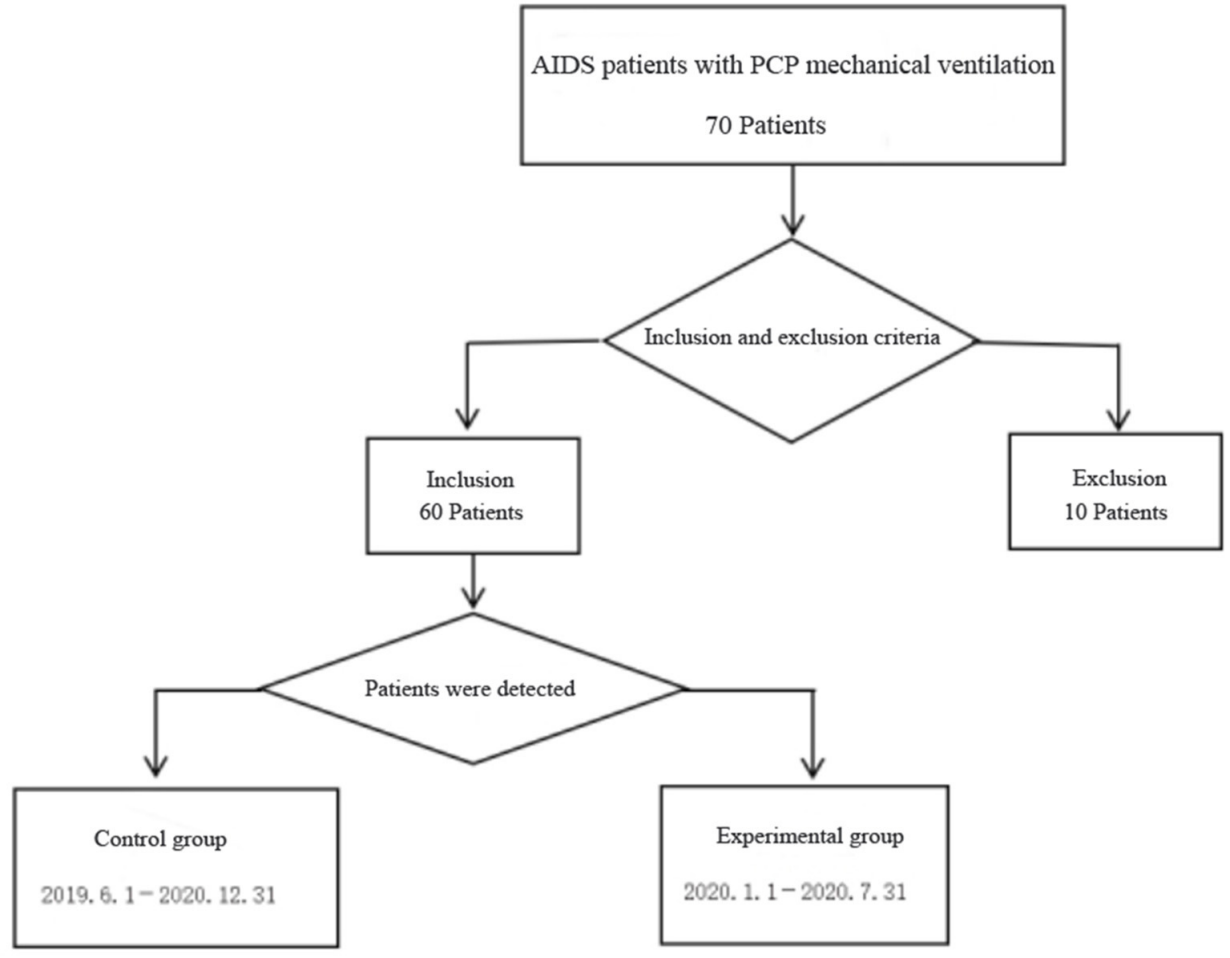
Flow chart.

All patients who met the criteria from 1 June 2019 to 31 December 2019 were selected as the control group. All patients who met the criteria from 1 January 2020 to 31 July 2020 were selected as the case group. See Figure 1 for the patient admission flow chart. This study has passed the ethics review of the Ethics Committee of Beijing You'an Hospital, and the ethics number is LL-2019-035-K.

## Methods and Variables

### Team Formation

All members of the team passed the collective training of respiratory therapists and had engaged in respiratory therapy for 3 months, with good professional skills. One of the nurses in charge who had obtained the ICU specialty nurse certificate was the team leader, and the members were composed of five ICU specialty nurses. Among them, there were two males and three females, with an average age of 30 years, average working life of 9.5 years, three nurses in charge and two nurses. The 12-h work system was implemented to achieve 24-h seamless connection and meet the various respiratory treatment needs of patients at any time.

### Nursing Plan

The scope of work of the respiratory team included management and maintenance of respiratory therapy equipment, ventilation parameters of respiratory support therapy patients, airway management, transport of critically ill patients, participation in the formulation of nosocomial infection protection measures in the ward, training of respiratory therapy expertise and operational skills and participation in the formulation of respiratory treatment norms.

The specific nursing plan is shown in Table 1.

**Table 1:** Comparison of nursing plans between the two groups.

	**Control group**	**Case group**
Operation mode	Doctor-led respiratory therapy	Respiratory therapy team-led respiratory therapy
Airway humidification	Single-servo heating mode	Double-servo heating control type respiratory humidifier device
Suction of airway secretions	On-demand sputum suction	On-demand sputum suction, fiberoptic bronchoscope suction
Nebulization treatment	Ordinary medical nebulizer	Vibrating mesh nebulizer
Vibration expectoration	Sputum excretion instrument, over turning and back patting	Sputum excretion instrument, over turning and back patting, prone position ventilation
Balloon management	Manual pressure gauge	Electronic airbag pressure gauge monitoring
Rehabilitation	Rehabilitation exercise according to the doctor's instructions	Individualized rehabilitation exercise plan

### General Monitoring Indicators

The general monitoring indicators include the patient's general condition (including gender, age, body mass index, APACHE II score, etc.).

### Observation Indicators

The observation indicators include time of invasive mechanical ventilation, the average length of hospitalization and the average hospitalization expenses of patients.

### Statistical Analysis

The Calculation of the Sample Size: A. With “Time Using the Ventilator” as the Outcome Indicator, the Most Clinical Difference (1–2 μ) Was Expected to be 10 H. The Standard Deviation Was nine and 12 H for the Case and Control Groups, Respectively. The Above Data Were the Results of Previous Clinical pre-Studies in the Department. Significance Level α Was 5%. Test Power of 1–β Was 90%. The Minimum Sample Size Was 17 Cases (34 Cases in two Groups) Calculated by PASS 11 Statistical Software. b. With the “Average Hospital Stay” as the Outcome Indicator, the Most Clinical Difference (1–2 μ) Was Expected to be 5 Days. The Standard Deviation Was Seven and 8 Days for the Case and Control Groups, Respectively. The Above Data Were the Results of Previous Clinical pre-Studies in the Department. Significance Level α Was 5%. The Test Power 1–β Was 90%. The Minimum Sample Size Was 28 Cases (56 Cases in two Groups) Calculated by PASS 11 Statistical Software. c. With “Average hospitalization Expenses” as the Outcome Indicator, the Most Clinical Difference (1–2 μ) Was Expected to be 2,000 Yuan. The Standard Deviation Was 1,530 and 1,320 Yuan for the Case and Control Groups, Respectively. The Above Data Were the Results of Previous Clinical pre-Studies in the Department. Significance Level α Was 5%. The Test Power 1-β Was 90%. The Minimum Sample Size Was 20 Cases (40 Cases in two Groups) Calculated by PASS 11 Statistical Software. Based on the Above three Calculation Results, two Were Finally Selected to Include 30 Patients, Respectively, With a Total of 60 Patients.

For Measurement Data, Those That Conformed to the Normal Distribution Were Represented by Mean ± Standard Deviation ($\bar{x}$ ± s). The Independent Sample *t*-Test Was Used for Comparison Between the two Groups, and the Median (IQR) Was Used for the non-Normally Distributed Data. The Comparison Between the two Groups Was Statistically analyzed by the Rank sum Test. The Statistics of the Count Data Were Expressed as the Composition Ratio (%), and the chi-Square Test Was Used for Statistical Analysis. *P* < 0.05 was Considered to be Statistically Significant. SPSS 21.0 Software Was Used for Statistical Analysis.

## Results

### General Information

A total of 60 patients were enrolled in this study, 30 in the control group, including 25 men (83.3%) and five women (16.7%); 30 in the case group, including 24 men (80.0%) and six women (20.0%). The basic data of the two groups were compared (see Table 2), and the difference was not statistically significant (*P* > 0.05).

**Table 2:** Comparison of general information of the two groups of patients.

**Group**	**Number of cases**	**Gender (cases)**		**Age (years)**	**Body mass index**	**APACHEII score (points)**
		**Male**	**Female**	**Mean ±standard deviation**	**Mean ±standard deviation**	**Mean ±standard deviation**
Control group	30	25	5	46.13 ± 15.91	24.62 ± 3.568	15.88 ± 2.17
Case group	30	24	6	45.94 ± 14.61	24.08 ± 4.825	15.41 ± 2.72
*t*-value/ χ^2^		0.111		0.049	0.578	0.762
*P*		0.739		0.961	0.565	0.449

### Comparison of Mechanical Ventilation Time, Average Length of Hospitalization, and Average Hospitalization Expenses Between the Two Groups

The comparison of mechanical ventilation time, average length of hospitalization, and average hospitalization expenses between the two groups were statistically significant (*P* < 0.05).

As shown in Table 3, since the establishment of the respiratory treatment team in our hospital, they have played a significant role in the respiratory system treatment of patients with AIDS complicated with PCP undergoing mechanical ventilation, shortened the mechanical ventilation time and the average length of hospitalization, reduced average hospitalization expenses and guaranteed the treatment effect of patients.

**Table 3:** Comparison of mechanical ventilation time, average length of hospitalization, and average hospitalization expenses between the two groups.

**Group**	**Number of cases**	**Mechanical ventilation time**	Average **length of hospitalization (d)**	**Average hospitalization expenses (yuan)**
		**(h)**		
		**(Median, range)**		
Control group	30	60, 36–200	29.2 ± 9.9	1,3184.5 ± 2,716.4
Case group	30	47, 20–188	23.1 ± 8.1	1,0103.7 ± 1,318.4
*P*		0.03	<0.01	<0.01
*T*			4.836	3.447

## Discussion

Mechanical ventilation is an important treatment for patients with AIDS complicated with PCP. The results of this study showed that the time of mechanical ventilation was significantly shortened, which was similar with the study of Pan et al. (11). The results may be related to the following factors. a. Specialized management of the airway: the respiratory treatment team was managed uniformly, which strengthened the systematicness and continuity of airway management. b. Prone position ventilation: prone position ventilation improves the lung ventilation blood flow ratio and matches the ventilation blood flow. It promotes dorsal alveolar recruitment, improves the ventilation blood flow ratio and enables good drainage of tracheal secretions due to gravity. This technique has been widely used in the clinical treatment of ARDS patients. Nurses must master how to effectively and safely change the patient's body position, ensure that the patient obtains sufficient oxygenation and ventilation, provide individualized nursing, closely monitor vital signs and other indicators, find and deal with all kinds of complications as soon as possible, stop in time in case of abnormalities and prepare all types of rescue equipment and drugs. c. Daily interruption of sedation (DIS) was proposed by Kress et al. in 2,000 (12); that is, reducing or interrupting the infusion of sedative drugs for patients with continuous sedation every morning until the patient can wake up and make corresponding actions according to instructions. For patients who cannot fully wake up, the goal is to increase blood pressure, heart rate and involuntary movement, which can help nurses evaluate the patient's condition in time, shorten the sedation time and avoid excessive sedation. d. The respiratory team used the PS-SBT method to evaluate the patient's weaning conditions so that the timing of weaning could be more accurately grasped. e. The implementation of early activities and respiratory function exercises: early breathing training can improve the respiratory function of mechanically ventilated patients, enhance the muscle strength and endurance of the respiratory muscles and relieve breathing difficulties.

There were few studies on respiratory therapy team. Chen Ye (13) mainly discussed the role of team model of joint respiratory therapist in preventing VAP in patients with mechanical ventilation. Pan et al. (11) Studied the working mode of building a nurse led respiratory therapy lung rehabilitation team in the lung rehabilitation of severe patients. Our research shows that the respiratory therapy team plays an important role in the treatment of AIDS patients complicated with PCP receiving mechanical ventilation.

Due to the heavy workload and unplanned work in traditional nursing care, it is difficult to timely observe, comprehensively evaluate and provide targeted nursing for patients' airways. However, in response to the above-mentioned problems, the professionally trained respiratory therapy team adopts an evidence-based collection of targeted nursing measures and carries out targeted nursing interventions based on conventional nursing to make up for the deficiencies of traditional nursing measures. The members of this team have specific clinical work experience, have been trained in respiratory therapy and have passed the hospital's certification to meet its respiratory therapy-related needs. Different from those who graduated with a traditional respiratory therapy major, the nurse-led respiratory therapy team can combine respiratory therapy with nursing, executing treatment more quickly, paying more attention to the implementation of integrated treatment measures, effectively monitoring and grasping the situation of patients and considering the tolerance of their treatment measures, rather than solving the clinical problems of patients one by one. At the same time, the team also serves as a bridge in the communication between doctors and nurses and chooses more suitable pulmonary rehabilitation programs for patients.

The nurse-led respiratory therapy team promotes the development of nursing professionals. Its successful establishment marks the path of professionalization and standardization of the nursing team in our hospital. At present, the team is carrying out various tasks in the entire hospital, and the professional ability of team members has continuously improved.At the same time, it has also been recognized by the medical team. It has played a homogenous management purpose in the respiratory treatment of the entire hospital.

This was a retrospective cross-sectional study. The limitations of this study included the limitations inherent in reviewing retrospective data. Although our data set was robust and associated with electronic medical records, there was the possibility of selection bias. In addition, it was a single-centre study. All cases in this research were in the same hospital and carried out by the same respiratory therapy team. In the next step, different respiratory therapy teams can participate in multiple hospitals to explore the wide range of applications of the results of this study.

## Conclusion

Although the respiratory therapy team can play a vital role in the treatment of patients with AIDS complicated with PCP undergoing mechanical ventilation, the construction and development of the respiratory therapy team, the selection of trainees, standardized training and certification, etc., are still problems that need to be solved urgently.

## Data Availability Statement

The original contributions presented in the study are included in the article/supplementary material, further inquiries can be directed to the corresponding author/s.

## Ethics Statement

The studies involving human participants were reviewed and approved by Ethics Committee of Beijing Youan Hospital. The patients/participants provided their written informed consent to participate in this study.

## Author Contributions

YG, SX, and LZ: conception and design of the research. YG, SX, BJ, and HG: acquisition of data. YG, SX, JY, and XL: analysis and interpretation of the data. YG, SX, XW, and LZ: statistical analysis. YG and SX: writing of the manuscript. LZ: critical revision of the manuscript for intellectual content. All authors contributed to the article and approved the submitted version.

## Conflict of Interest

The authors declare that the research was conducted in the absence of any commercial or financial relationships that could be construed as a potential conflict of interest.

## Publisher's Note

All claims expressed in this article are solely those of the authors and do not necessarily represent those of their affiliated organizations, or those of the publisher, the editors and the reviewers. Any product that may be evaluated in this article, or claim that may be made by its manufacturer, is not guaranteed or endorsed by the publisher.
